# A Case Series Demonstrating the Spectrum of Invasive Methicillin‐Sensitive *Staphylococcus aureus* (MSSA) Disease

**DOI:** 10.1155/crdi/8839477

**Published:** 2026-06-25

**Authors:** Elisée Mbombo, Mubarack Ayamie, James Makina, Doreen Phiri, Natasha Banda, Zainab Maluk, McPherson Mahala, Humphry Chisambiro, Nohakhelha Nyamulani, Franck Phoya, George Chagaluka, Patrick Mapemba, Emmie Mbale, David Kulapani, Alix N. Kamina, Criss K. Mjumbe, Josephine Langton, Yamikani Chimalizeni, Kondwani Kawaza

**Affiliations:** ^1^ Pathology Department, Kamuzu University of Health Sciences, Southern Region, Blantyre, Malawi; ^2^ Laboratory Department, Malawi-Liverpool Wellcome Research Program, Southern Region, Blantyre, Malawi; ^3^ Pediatrics and Child Health Department, Queen Elizabeth Central Hospital, Southern Region, Blantyre, Malawi; ^4^ Trauma and Orthopedics, Surgery Department, Queen Elizabeth Central Hospital, Southern Region, Blantyre, Malawi; ^5^ Emergency Medicine Department, Queen Elizabeth Central Hospital, Southern Region, Blantyre, Malawi; ^6^ School of Public Health, Université Officielle de Mbujimayi, Kasaï Oriental, Mbujimayi, Democratic Republic of the Congo; ^7^ School of Public Health, Université de Lubumbashi, Haut Katanga, Lubumbashi, Democratic Republic of the Congo

**Keywords:** disseminated staphylococcal infection, invasive MSSA disease, septic arthritis, skin lesions, *Staphylococcus aureus*

## Abstract

**Introduction:**

*Staphylococcus aureus*, although a common colonizer of skin and mucous membranes in humans, is associated with severe infections in both healthy and debilitated individuals. The mortality induced by invasive MSSA disease is high, especially in patients diagnosed late. In children, skin and joint manifestations are most common. Septic shock is a major contributing factor to early mortality. To date, reported cases of invasive MSSA disease in children are infrequent, especially in Sub‐Saharan Africa.

**Case Report:**

We present 4 cases of previously healthy children who presented with various clinical manifestations, but skin lesions and fever were common in all of them. They had multisystem involvement and eventually required aggressive multifactorial therapy. Antibiotic treatment was tailored to the results of antimicrobial sensitivity tests following blood culture. Though the hospital stay was quite long, intensive care and appropriate antibiotics contributed to a good prognosis.

**Conclusion:**

In children with invasive MSSA disease, early diagnosis and prompt management are key factors to improved prognosis. Skin lesions with tender joints in a febrile infant should raise suspicion for a staphylococcal infection. Intensive care and infection source control are the cornerstones of the management.

## 1. Introduction

*Staphylococcus aureus* is a pathogen well‐known to cause a wide spectrum of infections in children in developing countries, ranging from minor skin infections to life‐threatening systemic and disseminated disease with various clinical pictures [1]. The Centers for Disease Control and Prevention (CDC) estimates that 33% of the global population carries *S. aureus* in their nasal cavity, with other sites including the skin, the pharynx, and the gastrointestinal tract [2]. These infections can arise in otherwise healthy individuals or complicate the clinical course of a preexisting disease, accounting for high morbidity and mortality [3]. Septic shock is a substantial contributor to early mortality. In 2019, *S. aureus* alone was the first bacterial cause of death in 135 countries, inflicting 1,105,000 deaths [4]. The five stages in the pathogenesis of *S. aureus* infections are colonization, local infection, systemic disseminated infection and/or sepsis, metastatic infection, and toxinosis [5]. Invasive *Staphylococcus aureus* disease (ISAD), also called disseminated *Staphylococcus aureus* infection, often occurs without a defined portal of entry but causes substantial rates of mortality [6]. Their emergence is reportedly favored by host immunosuppression and antibiotic resistance [7]. The most commonly described focus of disseminated infection includes osteomyelitis, joint and deep soft tissue infections [8]. Complications such as glomerulonephritis, thrombophlebitis, and pulmonary embolism, even though less frequent, are still possible, and thus should not be overlooked by pediatricians [9]. The management of ISAD relies on swift diagnosis, timely and appropriate antibiotic therapy, prompt surgical drainage of effusions, cardiorespiratory monitoring, and supportive care [1].

Invasive methicillin‐sensitive *Staphylococcus aureus* (MSSA) disease has been better described in adults than in the child population. Henceforth, data in this population group are scarce [8], especially in Sub‐Saharan Africa, with only a few cases reported to date. Paradoxically, the higher prevalence of invasive disease in children and the reason why their prognosis is seemingly better than in adults remains a mystery [7]. We report 4 cases of ISAD in previously healthy children who presented with varying clinical pictures. This paper aims to pinpoint the clinical polymorphism of ISAD and highlight the need for early detection of the infection in order to initiate prompt management and improve patient prognosis. Invasive *S. aureus* disease is defined as involvement of at least two distant organs with the presence of gram‐positive cocci in clusters and/or growth of *S. aureus* from at least one normally sterile body fluid [10].

## 2. Case Presentation

### 2.1. Case 1

**FIGURE 1:**
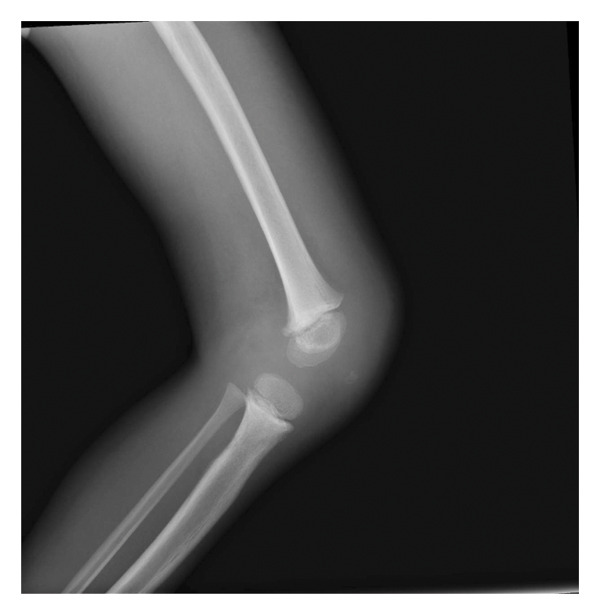
Osteosclerotic reaction, increased joint space, and soft tissue swelling in septic arthritis on the knee joint X‐ray.

A 9‐year old female patient referred from a health center was admitted with a 6‐day history of fever, shortness of breath, and inability to bear weight on her left leg. Chronologically, the patient first developed facial and left leg swelling with progressive inability to bear weight and fever. These were followed by pleuritic chest pain with progressive shortness of breath and a skin rash. The leg swelling was not associated with trauma or any bite and involved the right knee and both ankle joints. At the health center, a rapid malaria test was positive, and the patient was given a 3‐day course of lumefantrine–artemether (LA), but her fever was still spiking. There was no history of allergies or intoxication and no similar symptoms in the past. There was no known underlying comorbidity, and the other members of the family were all well. On physical examination, she was febrile, tachycardic, tachypneic, and normotensive with oxygen saturation at 90% in room air; her joints were swollen and warm to the touch with reduced range of motion, suggestive of septic arthritis. There was reduced chest expansion, reduced breath sounds, and vocal resonance on the mid and lower right lung zones, with basal crackles. Cardiovascular and abdominal examination were nonremarkable. Skin rash consisted of ill‐defined erythematous and edematous lesions, warm to the touch and tender, consistent with cellulitis over both legs. The patient was started on supplemental oxygen, and blood was drawn for laboratory workup. Complete blood count showed a low hemoglobin level of 6.8 g/dL, hematocrit of 20.1%, red blood cell count of 3.29 × 10^6^/μL, and an elevated white blood cell count of 18,690/μL. Arterial blood gas results were pH (7.382), pCO_2_ (32.3 mmHg), and pO_2_ (190 mmHg). Serum creatinine was 1.3 mg/dL. The HIV test was negative. Chest X‐ray revealed a right pleural effusion of moderate size. Ultrasound excluded both pericardial effusion and ascites. Knee joint X‐ray confirmed arthritis‐related lesions (Figure 1). The ceftriaxone start dose was given. Concurrently, supportive management consisted of intravenous fluids and blood transfusion. On the second day of admission, new symptoms of septic arthritis appeared in the left knee and left elbow joints. She was subsequently taken to the operating theater for arthrotomy and washout (including sampling). Pleural tap was also performed, and the sample was sent for culture. Blood, synovial fluid, and pleural fluid all grew *Staphylococcus aureus,* which showed sensitivity to chloramphenicol, ceftriaxone, cloxacillin, cotrimoxazole, clindamycin, erythromycin, gentamycin, meropenem, and tetracycline. With IV cloxacillin unavailable, and the susceptibility to ceftriaxone of most MSSA isolates in our institution, IV ceftriaxone was continued. Clindamycin was adjuncted to ensure synergy based on its antitoxin properties. On the course of this double regimen, the fever completely resolved, and skin lesions started improving, so antibiotics were continued for 14 more days. Cellulitis skin lesions were managed with daily cleaning and dressing. The patient ultimately required physiotherapy for mobility. Both repeat blood cultures were negative, confirming clearance of bacteremia. On Day 21 of admission and Day 17 following arthrotomy, she was discharged on oral cloxacillin for 6 weeks, and the guardian was instructed on the follow‐up plan, which consisted of oral cloxacillin and review visits at the nearest hospital. Since the patient was residing in a remote area, she was followed up at a local district hospital. The latest follow‐up at the district hospital revealed a complete functional recovery with full weight‐bearing and improved range of motion. However, the residual range of motion deficits were not objectively measured.

### 2.2. Case 2

A 5‐year‐old male patient, previously well, was referred to the pediatrics emergency unit from a peripheral health center for ongoing fever, shortness of breath, and left knee joint swelling for the previous 5 days. He was managed with artesunate for severe malaria, and ceftriaxone and metronidazole at the health center, but his condition was worsening. On examination, he was ill‐looking, pale, mildly jaundiced, febrile, tachycardic, tachypneic, and breathless, with oxygen saturation of 86% in room air. He had slightly reduced vocal resonance on his right chest, a massive splenomegaly, and a petechial skin rash on the abdomen. His swollen left knee and hip were warm and tender with limited range of motion.

**FIGURE 2:**
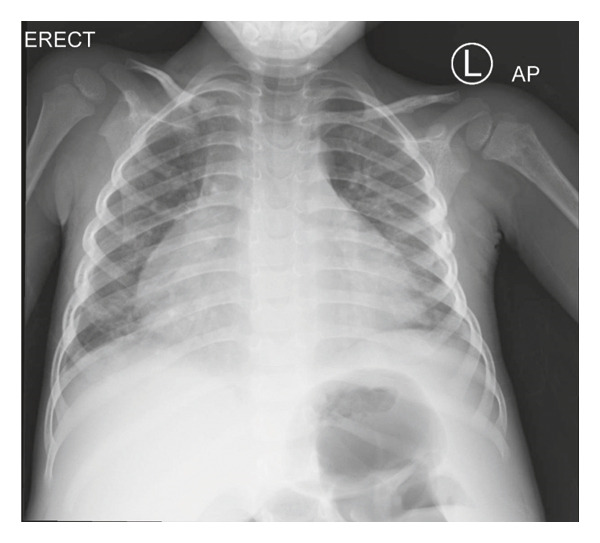
Right pleural effusion on a chest X‐ray.

A rapid ultrasound scan revealed a moderate right pleural effusion and an enlarged spleen, and chest X‐ray confirmed pleural effusion (Figure 2). No ascites was noted. As per laboratory results, initial complete blood count results showed low hemoglobin of 6.7 g/dL, low hematocrit of 21%, high white blood cell count of 15.28.10^3^/μL, and low platelet count of 13.10^3^/μL. His arterial blood gas findings were consistent with respiratory alkalosis. Serum electrolyte analysis showed mild hypokalemia, mild hyponatremia, and hypocalcaemia. The blood sample was sent for culture and antimicrobial susceptibility testing.

Initial medical management consisted of supplemental oxygen therapy, IV fluids, whole blood transfusion, an antistaphylococcal combination of flucloxacillin and clindamycin for a suspected ISAD, and continuation of malaria therapy with LA while awaiting surgical review once hemodynamic stability was achieved. Patient’s concurrent severe malaria, for which he had received a 24 h full dose of IV artesunate before referral, warranted a follow‐up treatment with an artemisinin‐based combination therapy such as LA as per the World Health Organization (WHO) 2021 Guidelines for malaria [11]. Nonetheless, on Day 3 postadmission, patient’s dyspnea worsened, and new joints were involved. Skeletal survey findings coupled with clinical features were consistent with septic arthritis involving the left hip, left knee, left ankle, right hip, and elbow joints. There was no lesion suggesting osteomyelitis on radiography. Repeat complete blood count showed leukocytosis with a WBC count raised at 21.2.10^3^/μL and a low platelet count of 46.10^3^/μL. Platelet transfusion was provided, and the patient was taken to the theater for arthrotomy and washout, as well as placement of a chest drain. Pleural fluid specimen, blood, and synovial fluid samples grew MSSA, which was sensitive to ceftriaxone, cloxacillin, chloramphenicol, erythromycin, clindamycin, cotrimoxazole, tetracycline, and gentamicin. The patient was then switched to a combination of ceftriaxone and clindamycin as per the results of antibiotic susceptibility testing. However, 2 weeks later, new foci of arthritis occurred in the right knee and left elbow, and a relapse in the left knee, which prompted a second arthrotomy and washout. Physiotherapy was started after the second surgery for mobility purposes. Repeat blood cultures were negative, and antibiotic therapy was maintained for 42 days with satisfactory evolution. No more arthritis relapse was noted, the chest drain was removed, and the patient was discharged on oral cloxacillin with physiotherapy on Day 47 after admission.

### 2.3. Case 3

A 10‐day‐old male patient was admitted with fever, high‐pitched cry, and poor feeding for 4 days, followed by an eruption of macular skin rash on the chest and the abdomen the next day. Diarrhea and vomiting were also reported. Oral paracetamol was given, but with only a mild relief of the fever. He was born at term via spontaneous vaginal delivery to a 25‐year‐old primigravida with a birth weight of 3.8 kg. The pregnancy had been uneventful; the mother had fully attended antenatal clinic and was up‐to‐date with her immunization schedule. Both parents were known to be healthy, and the mother tested negative for HIV, syphilis, and hepatitis B. There was no history of blood transfusion, no known allergies, no medication administered, and no previous hospital admission.

**FIGURE 3:**
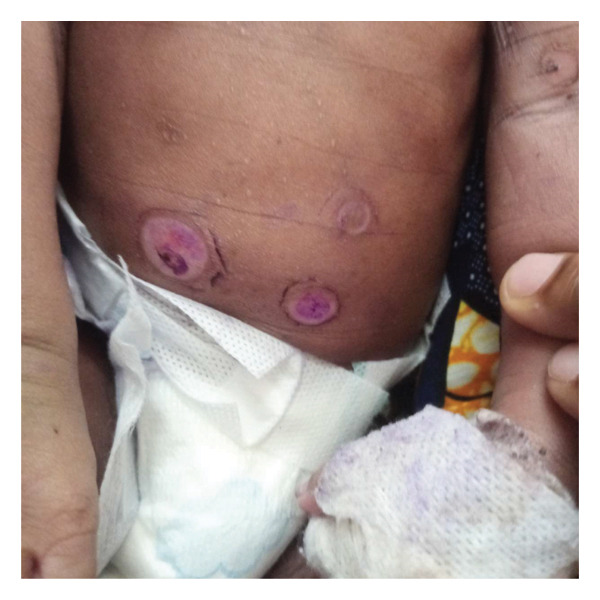
Healing scalded skin lesions in a neonate (source: authors).

On the presentation, the patient was profoundly shocked with cold extremities and a weak pulse, and tachypneic and tachycardic with oxygen saturation of 88% in ambient air, but the chest was otherwise clear; his level of consciousness was depressed but with no focal neurological signs. Although his nutritional status looked fair, he had lost 32% of his body weight (2.6 kg on presentation versus 3.8 kg at birth). The fluid deficit formula (percentage of dehydration × body weight) was used to calculate his fluid deficit. Considering patient’s initial weight of 3.8 kg and an estimated 15% dehydration, his computed fluid deficit was 0.57 L [12]. Otho–rhino–laryngological and abdominopelvic examination was nonremarkable. There was no lymphadenopathy. Skin lesions were made of large macular rash superseded by necrotic epidermolysis, indicating scaled skin (Figure 3). The rest of the physical examination was normal. Random blood sugar was 141 mg/dL. The malaria rapid test was negative. Complete blood count revealed WBC count of 19,400/μL, red cell count of 6.47.10^6^/μL, hemoglobin of 19.4 g/dL, hematocrit of 70.3%, and low platelet count of 144,000/μL. Arterial blood gas showed compensated respiratory acidosis with pH (7.304), pCO_2_ (53.8 mmHg), pO_2_ (50 mmHg), and HCO_3_ (26.7mEql/L). Serum sodium was high (182 mmol/L), but other electrolytes were within normal range. Blood urea nitrogen and serum creatinine showed increased values, 42 mg/dL and 5.6 mg/dL, respectively, indicative of acute kidney injury. Cerebrospinal analysis was nonremarkable. The patient was suspected to have toxic shock syndrome complicating staphylococcal scalding skin syndrome, based on the CDC diagnostic criteria for toxic shock syndrome [13]. He was admitted to the pediatric intensive care unit (PICU) and managed with intravenous fluids, adrenaline infusion, furosemide, ceftriaxone, and clindamycin. The cautious management of this hypernatremic dehydration was carried out with initial intravascular fluid restoration with NaCl 0.9% 20 mL/20 min repeated twice, and ultimate correction was achieved slowly over 72 h with 5% dextrose + half‐normal saline (with 20 mEq/L KCl) as per standard practice. The choice of ceftriaxone for initial management was based on the local antimicrobial susceptibility profile of MSSA, which is highly susceptible to ceftriaxone. Respiratory support with continuous positive airway pressure (CPAP) was provided for 3 days. The hypernatremia and acidosis persisted for almost 5 days before normalizing, Band UN and serum creatinine started coming to normal on Day 3. A feeding plan was set up in parallel to ensure the patient′s adequate alimentation. Once hemodynamically stable, the patient was transferred to pediatrics high dependence unit (PNHDU) on Day 7, but was still having spikes of fever and extending skin lesions. The malaria parasite screen was negative. The first BC did not show any growth. A repeat BC was performed, growing MSSA, which was also tested on meropenem and found to be sensitive. The severity of the disease prompted escalation to IV meropenem. Once switched to meropenem, the patient’s condition started improving as demonstrated by defervescence and healing of skin lesions, as well as weight gain. The follow‐up blood culture was negative. On Day 10 of admission in the PNHDU ward, the patient was discharged on oral cloxacillin and scheduled for a review, completing a 17‐day hospital stay.

### 2.4. Case 4

**FIGURE 4:**
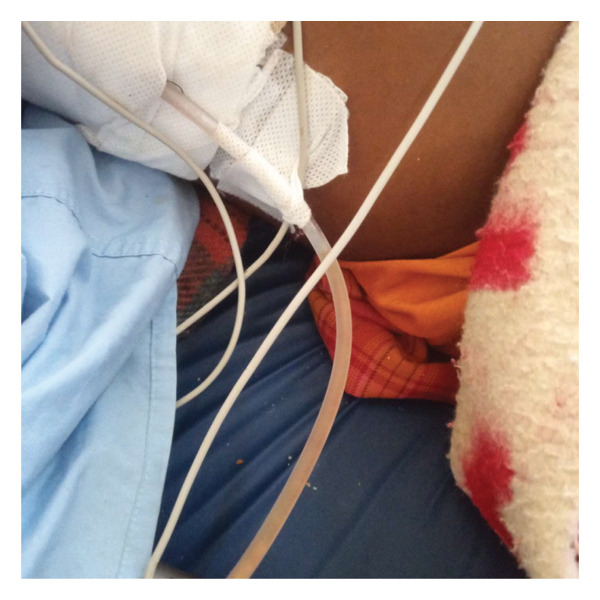
In‐situ chest tube draining pleural fluid (source: authors).

A 13‐year old male patient presented with fever, cough, skin rash, pleuritic chest pain, and abdominal swelling for 3 days. He reported falling from a height 7 days earlier, but with no apparent traumatic injury. On his examination, he was noted to be ill‐looking, dyspneic, febrile, pale, irritable, and mildly confused with a Glasgow Coma Scale of 13/15. He had coarse crackles over the right lung zone and reduced vocal sound, with dullness to percussion consistent with a pleural effusion. His abdomen was mildly distended with shifting dullness, suggesting ascites. Neurologic and musculoskeletal system examination was otherwise normal. Body temperature was 38.6°C, respiratory rate was 58 bpm, and spO_2_ was 87% in ambient air. Chest X‐ray showed right pleural effusion, while abdominal ultrasound confirmed moderate ascites. Blood and pleural fluid samples were sent for culture, and the patient was resuscitated with fluids and blood transfusion, and also started on empiric IV ceftriaxone. A chest drain was placed, while ascites was managed conservatively (Figure 4). Both blood and pleural fluid culture grew MSSA, which showed sensitivity to some available antibiotics, including ceftriaxone, meropenem, and amikacin. Thus, ceftriaxone was continued until Day 14. Upon clinical improvement, he was switched to oral flucloxacillin and started on chest physiotherapy. Repeat blood cultures were all negative. The drain was eventually removed, and the patient was discharged on Day 24 on oral flucloxacillin.

## 3. Discussion

Invasive MSSA disease includes a set of clinical manifestations secondary to multiorgan invasion, ranging from cellulitis to more severe and deep‐located infections such as sepsis, toxic shock syndrome, endocarditis, and osteomyelitis [14]. Mortality associated with invasive disease in children is high, 30%–35% [15]. In pediatrics, the commonest complications are cutaneous and osteoarticular infections [8]. In cases reported, patients presented with at least two systems involved. Even though there was no recorded mortality, the morbidity was so high that patients required critical care. Unlike many other pathogens, *S. aureus* has the propensity to cause severe infections in otherwise healthy individuals [16]. As for *S. aureus* strains, MSSA is reported to be the most common etiological agent of disseminated infection in spite of the rise of resistant strains [17]. None of the isolated strains in reported cases was methicillin‐resistant (methicillin‐resistant *Staphylococcus aureus* [MRSA]), which correlates with current literature.

Some research revealed that a bacteria–fungi interaction, through biofilm formation on epithelium, is a sine qua non for systemic infection, even though the mechanism by which bacteria spread from epithelial surfaces to internal organs is still unknown [6]. Major risk factors for disseminated staphylococcal infection include age (with infants and elderly at higher risk), additional comorbidities, presence of indwelling medical devices, intravenous drug use, and low socioeconomic status [18]. In addition to that, risk factors for neonatal staphylococcal infection are prematurity, low birth weight, and extended hospital stay [1]. Some existing data suggest that in the pediatric population, invasive disease is more frequent in infants less than one‐year‐old [19]. To our knowledge, none of the cases described here had a known comorbidity, and there was no history of recent hospitalization, which could have put them at risk of healthcare‐associated *Staphylococcus aureus* infection. Moreover, disseminated staphylococcal infection seems to involve all age groups of children with no predominant category. A large‐scale study may be needed to provide more insights into which age group might be most concerned. In reported Case 3 of neonatal infection, the patient did not have any of the above‐listed risk factors for neonatal disease, as he was a term baby, weighing 3.8 kg at birth, with no history of previous hospitalization nor had he any prior indwelling device; antepartum and/or intrapartum risk factors ought to be considered. It should be recalled that almost one‐half of patients who were documented to have ISAD do not have an identifiable avenue of entry [20].

Osteomyelitis is reportedly the most common initial focus of ISAD, being the starting point in roughly 32.4%–62% of patients as per the reviewed series [17]. However, in cases described here, septic arthritis was the most frequent initial focus. In children, other frequent foci of dissemination include cellulitis, arthritis, endocarditis, and pneumonia. Thromboembolism and glomerulonephritis are seldom [21]. Other less common manifestations in children include staphylococcemia‐related renal failure and septic thrombosis [8]. In 8%–15% of the patients, hematogenous spread from primary foci may lead to secondary metastatic foci weeks to months after the primary infection, which could make the source control more challenging [22]. In Case 1, septic arthritis was the primary focus, with subsequent sepsis and hematologic disorders. Cellulitis, pneumonia, and associated pleural effusion were secondary foci of disseminated infection. Case 2 displayed a similar clinical presentation, but with an electrolyte imbalance in addition. This patient had relapsing septic arthritis. Deep venous thromboses were suspected to be the source of those recurrences. Riascos‐Pinchao et al. underlined the contribution of deep venous thromboses in the occurrence of recurrent septic arthritis [8]. Furthermore, *S. aureus* is known to promote venous thrombosis [23]. Skin lesions were the first apparent lesions in Case 3. Skin manifestations were consistent with staphylococcal scalded skin syndrome (SSSS) and were complicated by toxic shock syndrome. Hematological disorders and electrolyte imbalances were present as well. Locally, electrolyte derangement in neonates, especially hypernatremic dehydration, could be favored by compounding factors such as delay in lactation, ongoing fluid losses, and delay in seeking medical attention (often related to cultural or religious misbeliefs, unavailability of ambulance system, and inexperience of some primiparas in recognizing symptoms of neonatal disease). Some of these factors accounted for the extreme weight loss seen in Case 3. All patients presented with skin lesions. SSSS typically affects neonates and young children following localized infections, yet it may also occur in older patients with severe infections such as pneumonia, septic arthritis, and pyomyositis. It is mediated by Exfoliative toxins A and B, which cause symptoms. If SSSS is not promptly diagnosed, patients are at risk for other infections, in addition to sepsis and renal failure [24]. However, the prognosis of SSSS in young children is excellent in general [25]. Patient in Case 3 presented SSSS, probably following neonatal sepsis, which developed into toxic shock and acute kidney injury. With the patient′s septic condition persisting long after resolution of toxic shock, a concurrent septic shock should not be overshadowed. However, the outcome was good as the patient’s condition improved with therapy. Considering presentations in Cases 1 and 2 versus Cases 3 and 4, invasive MSSA disease can take various clinical pictures, as reported by Hon et al. [26].

Early diagnosis is fundamental to the successful management of invasive MSSA disease [27]. Patient in Case 3 presented earlier, before pleurisy and septic arthritis lesions developed, while those in Cases 1, 2, and 4 presented late with more disseminated infection. There is a constant question over the significance of blood culture results, as positive results with *S. aureus* are sometimes underestimated or misinterpreted as contamination. As a general rule, blood cultures positive for *S. aureus* are always regarded as a clinically significant finding and should lead to appropriate treatment. Blood culture contamination with *S. aureus* is a rare phenomenon (< 5%), and considering the high mortality and increased risk of severe complications associated with *S. aureus* systemic infections, rapid therapy is strongly recommended [28, 29]. Patients require a thorough clinical examination followed by corresponding investigations to localize the source and initiate a targeted therapy, and subsequently improve the prognosis [30]. Source control also helps prevent secondary septic metastases [31]. When antibiotic susceptibility patterns are not yet known, empiric antibiotic therapy consists of an intravenous antistaphylococcal penicillin or cephalosporin in children aged over 3 months, with the addition of gentamicin in those aged under 3 months. An oral regimen may also be considered depending on the bioavailability of the antibiotic chosen and the data from clinical and laboratory assessment. Once susceptibility results are known, de‐escalation and monitoring can be implemented. In MSSA strains, vancomycin monotherapy is less effective [27, 30]. Early switch‐over to oral regimen, basically after 10–14 days, should be considered once patient’s clinical condition improves. Additive measures are aimed at tackling life‐threatening conditions and may include prompt optimization of oxygen delivery, optimizing hematocrit (> 30%), and maintaining optimal central venous pressure (80–120 mmHg) with aggressive fluid resuscitation and early use of inotropes [10]. There is still debate over the duration of parenteral antibiotic treatment because of persistence, recurrence, and appearance of new foci of infection for a quite prolonged period of time. Nevertheless, the consensus is in favor of prolonged (4–6 weeks) parenteral therapy in patients with deep‐seated infections [32]. In our setting, considering the relatively high sensitivity of *S. aureus* to ceftriaxone, this remains the first‐line empirical antibiotic in invasive MSSA disease. Its continuation or discontinuation depends on antimicrobial susceptibility testing results. Carbapenems are used in the third line. Clindamycin is often adjuncted to ceftriaxone for its antitoxin properties. Clindamycin is a protein synthesis inhibitor which works by inhibiting ribosomal protein synthesis, leading to a reduction in the production of several staphylococcal exotoxins, which could theoretically reduce pathogen virulence and improve clinical outcome in *Staphylococcus aureus* bacteremia [33]. A randomized controlled trial involving 34 patients showed zero mortality (0/17) in the group of standard antistaphylococcal drug with clindamycin adjunct compared to 24% mortality (4/17) in the group taking standard therapy alone [34]. According to several reports, adherence to treatment can reduce mortality by up to 50% [35, 36]. However, mortality rate can remain as high as 20%–30% even in developed countries despite effective antibiotic treatment and source control strategies [37]. Thus, there is still a need for a prospective controlled study to address the issues of the appropriate duration of antibiotic therapy and effective adjuvant measures in invasive MSSA disease.

Patients need to be followed up, and all organ systems must be meticulously evaluated. The avenue of entry should be precisely traced and suppressed. The source focus should also be identified, as an unrecognized source can act as a continuum of seeding, definitely leading to future complications [14]. /The occurrence of septic shock and the need for mechanical ventilation carry a poor prognosis [1].

## 4. Conclusion

Invasive MSSA disease is a complex entity, more than just an infection, whose management requires a multidisciplinary approach. The disease is clinically polymorphic and displays various clinical presentations that should be readily recognized, as early diagnosis is the key to successful management. In children, skin and joint manifestations are common, and so staphylococcal infection must be included in the differential if a febrile child presents with skin lesions. Healthy individuals are as vulnerable to disseminated staphylococcal infections as debilitated patients. All patients with clinical suspicion of disseminated disease should be urgently hospitalized for intensive care of any emergencies. Timely initiation of aggressive support treatments and antibiotics and adequate source control are crucial for an optimal clinical outcome.

## Funding

This research did not receive any funding.

## Consent

Written informed consent was obtained from the parents for the publication of these cases and any accompanying pictures.

## Conflicts of Interest

The authors declare no conflicts of interest.

## Data Availability

Data sharing is not applicable to this article as no datasets were generated or analyzed throughout this research.
